# Etiology, distribution, treatment modalities and complications of maxillofacial fractures

**DOI:** 10.4317/medoral.19077

**Published:** 2013-12-07

**Authors:** Nathalie Pham-Dang, Isabelle Barthélémy, Thierry Orliaguet, Alain Artola, Jean M. Mondié, Radhouane Dallel

**Affiliations:** 1 Clermont Université, Université d’Auvergne, BP 10448, F-63000 Clermont-Ferrand; 2 Inserm, UMR1107, Trigeminal pain and Migraine F-63000 Clermont-Ferrand; 3 CHU Clermont-Ferrand, Service de Stomatologie et Chirurgie Maxillofaciale; 4 CHU Clermont-Ferrand, Service d’Odontologie, F-63003 Clermont-Ferrand, France

## Abstract

Purpose: This study evaluated the trends and factors associated with maxillofacial fractures treated from 1997 to 2007 in the Oral and Maxillofacial Surgery Department of the Clermont-Ferrand University Hospital. Material and Methods: This study included 364 patients of which 82% were men and 45%, 20-29-years old. The etiology, anatomical distribution, treatment modality and complications of maxillofacial fractures were examined. Results: Overall, interpersonal violence, traffic accidents and falls were the most common mechanisms of injury. There was a decreasing trend in traffic accidents and increasing one in falls as a cause of fracture over the 11-years period of this study. Young male patients were preferentially victim of interpersonal violence and traffic accidents, while middle-aged ones were of falls and work-related accidents. Middle-aged female patients were preferentially victim of traffic accidents and interpersonal violence, while older ones were of falls. And the number of fractures per patient varied according to the mechanism of injury: low after work-related accidents and high after traffic accidents. About two-third of fractures involved the mandible. Most of these mandibular fractures were treated by osteosynthesis with or without intermaxillary fixation, with the proportion of the latter increasing over time. There were very few postoperative infections and only in mandible. Conclusions: Maxillofacial fractures predominantly occur in young men, due to interpersonal violence. There is nevertheless an increasing trend in falls as a cause of fracture, especially in female patients, consistent with the increasing trend in presentation of older people. Most maxillofacial fractures involve the mandible and there is an increasing trend in treating these fractures by osteosynthesis without intermaxillary fixation. Antibiotic prophylaxis associated with dental hygiene care can be indicated to prevent postoperative infections.

** Key words:**Maxillofacial fractures, Epidemiology, Trends, Influencing factors, Fall, Age, Gender, Antibiotic prophylaxis.

## Introduction

The epidemiology and characteristics of maxillofacial fractures have now been described in many regions from around the world. The epidemiology of maxillofacial fractures appears to vary in the mechanism, severity and cause of injuries from one country to another and even within the same country (1). This suggests that many factors including socioeconomic and cultural conditions may locally influence the incidence of maxillofacial fractures. Such factors need to be identified. Collection of epidemiologic data regarding maxillofacial fractures, on the one hand, provides insight into the behavioral patterns of people from different regions. On the other hand, it is pivotal for evaluating existing preventative measures and designing new methods for preventing injuries (1).

There is little information regarding the epidemiology and characteristics of maxillofacial fractures in France (2). Moreover, estimates of trends in the factors associated with these fractures are not available. The purpose of this study was (i) to describe the etiologies, anatomical distribution, treatment modalities and complications of maxillofacial fractures and (ii) to examine trends in these factors in patients who were surgically treated from January 1997 to December 2007 at the Oral and Maxillofacial Surgery Department of the Clermont-Ferrand University Hospital. The city of Clermont-Ferrand is a medium size city (141 000 inhabitants) located in the centre of France, in the department of Puy-de-Dôme, part of the French region Auvergne (1.3 million inhabitants).

## Material and Methods

We conducted a retrospective study involving adult (? 18 years) patients with maxillofacial fractures who were surgically treated under general anesthesia from January 1997 to December 2007 at the Oral and Maxillofacial Surgery Department of the Clermont-Ferrand University Hospital (Clermont-Ferrand, France). Only patients who were transported directly to our department were included in this study. Were excluded patients who were transferred only subsequently to our department, that is (1) patients with large skin or blasted lesions (n = 6), and (2) patients with major facial trauma, including naso-orbital-ethmoidal (n = 2) and Le Fort fractures (n = 12). Because of other major traumatic injuries or neurological complications, such patients received initial treatment in another department, including the intensive care unit. They were only subsequently transferred to our department where definitive maxillofacial trauma care could be delivered.

Data were collected from the clinical notes and surgical records of each patient using a standardized, specifically designed form. Documented data are: 1) age and gender; 2) the cause of the trauma (traffic accident, interpersonal violence, work- or sport-related accident, falls), 3) the anatomical location of the fractures (angle, condyle, body, symphysis, ramus, zygomaticomaxillary complex, orbital floor, zygomatic arch), 4) time between trauma and treatment, 5) the type of treatment and 6) the postoperative complications.

Fractures were diagnosed with conventional radiography (dental panoramic radiography or Hirtz’s view X-ray or sinus X-ray) and maxillofacial computed tomography and segment displacement evaluated based on both clinical and image examination. Fractures were thus classified as displaced or not.

The complications that were taken into account are: 1) infection (inflammatory signs and suppuration from the fracture site, whether or not a treatment was required); 2) delayed bone healing (lack of consolidation after 6 weeks or more) and 3) exposed fixation material.

Treatments of mandibular fractures include: 1) intermaxillary fixation, using Dautrey arch and 0.4 mm stainless steel wire, with close reduction of the fractures, the patient being left in centric occlusion for 45 days; 2) osteosynthesis through an intraoral approach, using monocorticale screws and plates; or 3) combined osteosynthesis and intermaxillary fixation, the period of centric occlusion being reduced to 30 days. The type of treatment was selected by the surgeon according to the type of fracture, the characteristics of the patient and the need for rapid jaw mobility. Symphysial and mandible body fractures were always stabilized using two plates through an intra-oral approach. Angle fractures were treated with one plate fixed on the external oblique line through an intra-oral approach or, if not possible, a trans-oral one. Plates are made of 1.0 mm-thick pure low-grade titanium (Modus® system) stabilized with 7.0 mm-long, 2.0 mm-diameter screws.

The treatment of lateral midfacial fractures was performed after a delay of several days to allow clinical assessment after the oedema had reduced. Patients with a fracture of the zygomaticomaxillary complex or orbital floor were assessed for ocular functions (ophthalmologic consultation and Lancaster test). Lateral midfacial fractures were reduced using a Ginestet’s hook or Kilner’s lever. If needed, such reduction was stabilized by osteosynthesis using plates fixated by 5 mm long screws. When orbital soft tissue was entrapped, the fracture of the orbital floor was repaired by covering the orbital floor defect with PDS 0.25 (polydioxanon, Ethicon, Johnson and Johnson) via a subciliary approach.

All patients received prophylactic antibiotic. Patients with mandibular fractures received an oral antibiotic therapy [amoxicillin + clavulanic acid 3×1 g/day or clindamycin 3x300 mg/day in patients allergic to penicillin] until the day of surgery and for 5 days after surgery. In addition, we systematically inserted a nasogastric tube for enteral feeding to improve nutrition and protect healing for 5 days. Patients with lateral midfacial fracture received a per-operative parenteral [amoxicillin + clavulanic acid 2 g or clindamycin 600 mg in patients allergic to penicillin] and oral antibiotic [amoxicillin + clavulanic acid 3×1 g/day or clindamycin 3x300 mg/day in patients allergic to penicillin] for 5 days after surgery.

Post-operatively, patients received either paracetamol 1000 mg, alone or with 60 mg codeine, 3 times/day for 5 days. Teeth were brushed and cleaned with water jet toothbrush twice a day. Patients were also given chlorhexidine mouth rinse three times a day.

Results are expressed as mean ± Standard Error of the Mean (SEM). Statistical analysis was performed using Student’s t-test, one-way analysis of variance (ANOVA) followed by a post hoc Student-Newman-Keuls test or chi-square test. Trends over the study period were assessed by using (1) linear regression analysis and (2) comparing means within two 4-years periods, at the beginning (from 1997 to 2000) and end (from 2004 to 2007) of the study period. The level of significance was set at *P* < 0.05.

## Results

-Patients

**Figure 1:**
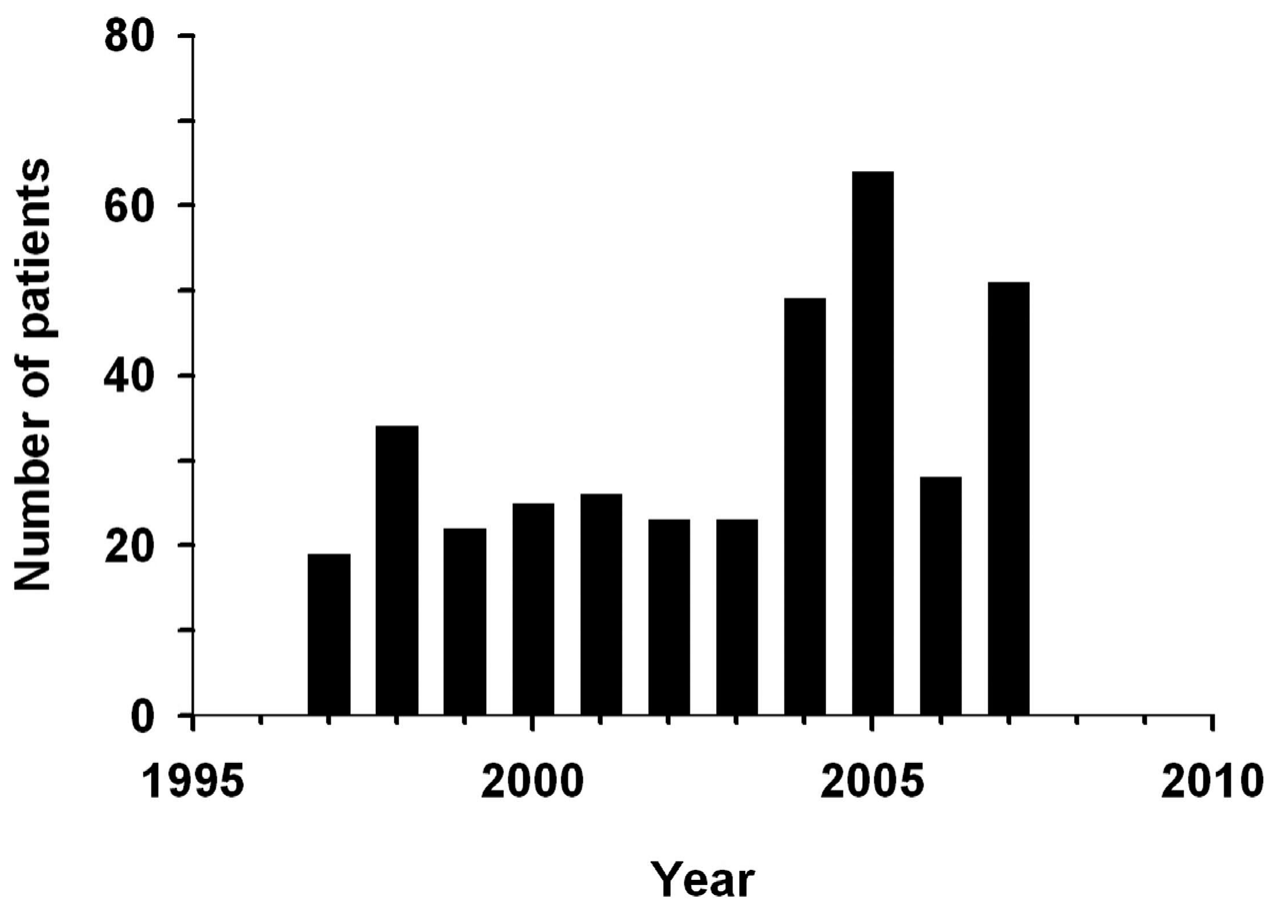
Annual incidence of fractures.

A total of 364 adult patients with maxillofacial fractures were surgically treated at the Oral and Maxillofacial Surgery Department of the Clermont-Ferrand University Hospital between January 1997 and December 2007. The annual incidence of maxillofacial fractures significantly increased over this time (Fig. 1): from 25 ± 3 to 48 ± 7 in the 1997-2000 and 2004-2007 4-years groups (referred to as early and late 4-years groups in the remaining of the paper; see Material and Methods), respectively.

**Figure 2:**
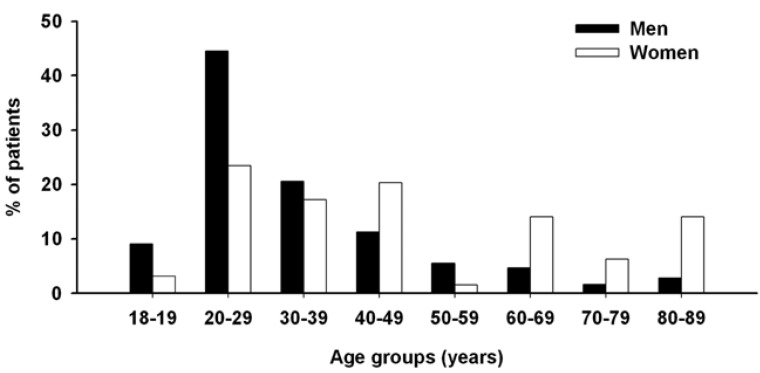
Age and gender distribution of patients.

Surprisingly, though the number of patients admitted in the department per year had nearly doubled over the 11-years period, patient demographic profile had remained rather consistent. Overall, 300 (82%) patients were male. Such sex ratio remained steady over time (85 ± 3 and 82 ± 2% in early and late 4-years groups, respectively). The overall age of patients was 34.0 ± 0.9 years (range 18 - 89 years) and the predominantly affected age group was between 20 and 29 years old, including 162 patients (45%) (Fig. 2). Female patients were significantly older than male ones (46.6 ± 2.7 years, range 18 – 83 years compared with 31.5 ± 0.7 years, range 18 – 89 years; *P* < 0.0001). Interestingly, whereas the mean age of male patients remained remarkably steady (30.2 ± 2.4 and 32.4 ± 0.9 years in early and late 4-years groups, respectively), that of female patients tended to increase, though not significantly (*P* = 0.11), over time (from 34.4 ± 5.9 to 47.8 ± 6.4 years in early and late 4-years groups, respectively). Overall, alcohol and tobacco use was found in 21% of patients, predominantly in male ones (88%). There was a trend (*P* = 0.037) towards increasing tobacco use (from 13 ± 1 to 24 ± 3% of patients in early and late 4-years groups, respectively).

-Causes of injury

The main causes of fractures in the overall population of patients were: interpersonal violence (39%, n = 143), traffic accidents (24%, n = 89), falls (20%, n = 73), sport- (12%, n = 45) and work-related accidents (4%, n = 14). Interpersonal violence was by far the most common (55%) cause of fractures in patients who used alcohol and tobacco. There was a significant (*P* = 0.022) reduction in traffic accidents as a cause of fracture over the 11-years period (from 33 ± 3 to 18 ± 2% in early and late 4-years groups, respectively). Conversely, the overall fall rate increased over time (*P* = 0.026; from 17 ± 2 to 22 ± 1% in early and late 4-years groups, respectively). Interestingly, such an increase predominantly occurred in female patients as it was highly significant in female (*P* = 0.003) but not in male (*P* = 0.092) patients.

**Figure 3:**
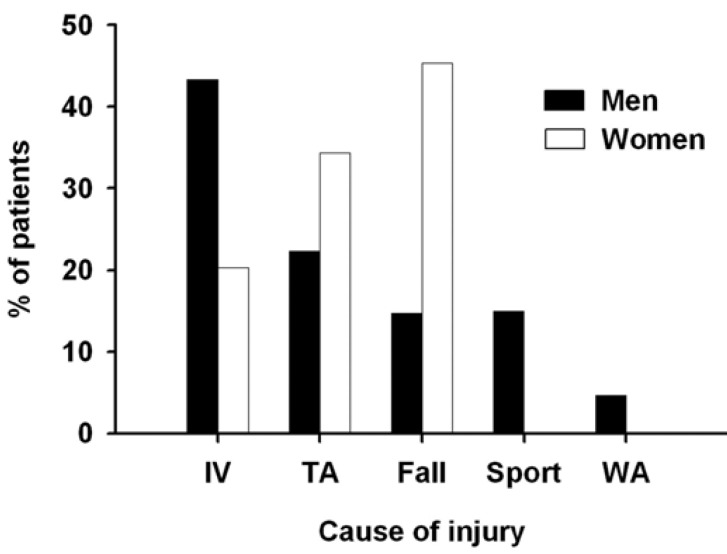
Gender distribution of the cause of fractures.

**Figure 4:**
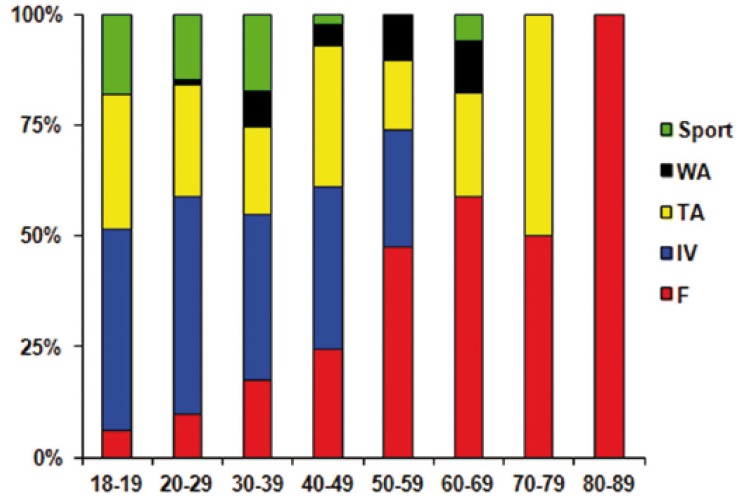
Age distribution of the cause of fractures.

The etiology of fractures significantly varied (*P* < 0.001;  Table 1) with gender (Fig. 3) and age (Fig. 4). Thus the main cause of fractures was interpersonal violence in male (43 %) and falls in female (45%) patients. Moreover, young male patients (~ 30 years) were preferentially victim of (with decreasing incidence) interpersonal violence, traffic accidents, and sport-related accidents whereas middle-aged ones (~ 40 years) were of falls and work-related accidents. In middle-aged female patients, the main causes of fractures were (with decreasing incidence) traffic accidents and interpersonal violence but, in older (~ 60 years) ones, falls. There was no association between the use of alcohol and the mechanism of fractures.

**Table 1:**
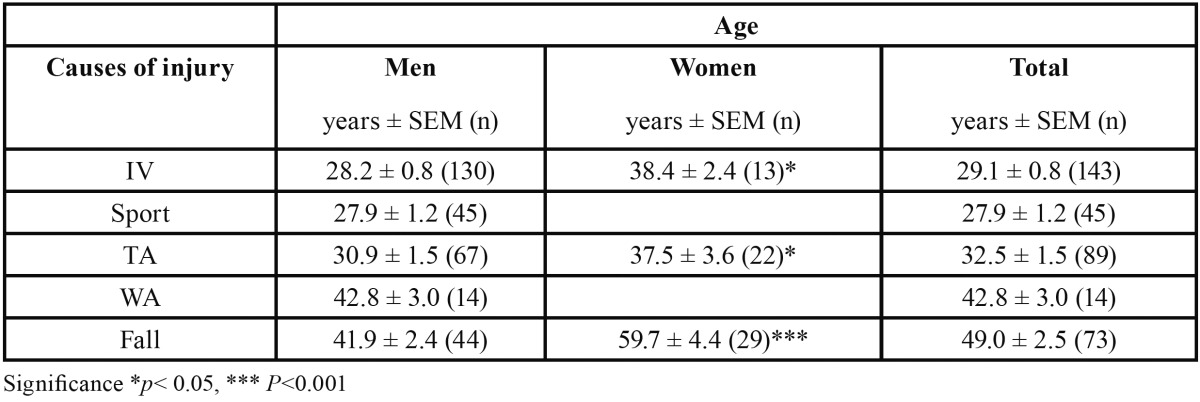
Age and gender of patients as related to the cause of injury.

-Fractures pattern

Most of the fractures involved either the mandible or the lateral midfacial region with a strong predominance of the former (236 and 114 cases, respectively; that is, 65% and 31% of total patients, respectively). In only fourteen patients (4%) were both the mandible and lateral midfacial region broken. There was no association between the use alcohol and location of fractures. Fifty four per cent (54%) of the mandibular fractures were unilateral, 38% bilateral and 5% in the middle mandible. Of the 209 unilateral fractures, 80 were on the right side and 129 on the left one.

**Figure 5:**
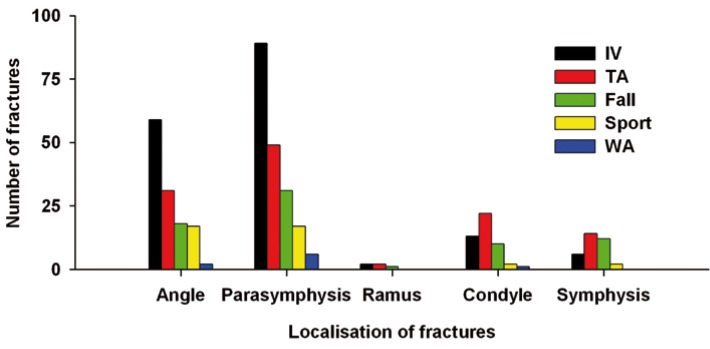
Number of fractures per patient according to the cause of fractures.

**Figure 6:**
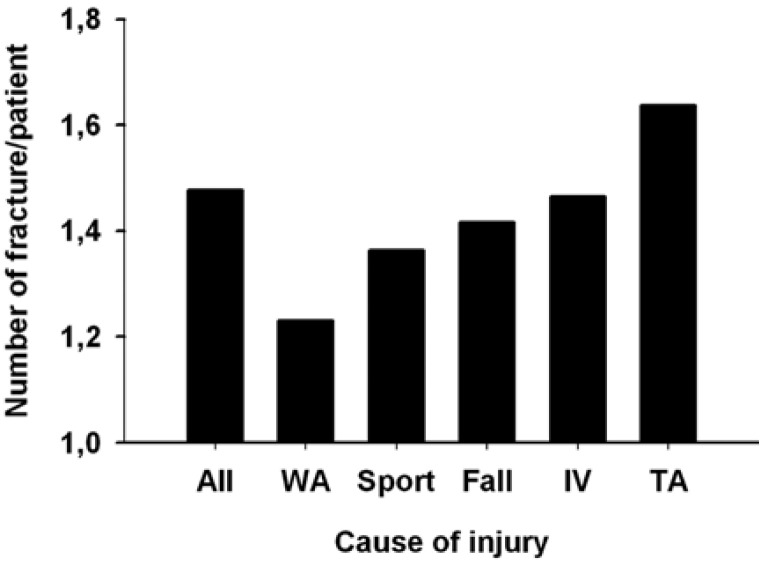
Time taken for repair of mandibular and lateral midfacial fractures.

Mandibular fractures were mostly unifocal (60%) or bifocal (32%), triple fractures being very occasional (8%). Of unifocal fractures, 71 involved the body, 26 the angle and, 20 the symphysis. Double fractures included usually both the body and angle (n = 80) and, less frequently, either body or angle with the symphysis, condyle or ramus. The most common type of triple fracture associated the condyle, body and angle. Unifocal fractures were equally caused by interpersonal violence, traffic accidents and falls. The average number of fractures per patient was 1.48 (Fig. 5). It varied according to the mechanism of injury (Fig. 6): low (1.21) after work-related accidents and high (1.64) after traffic accidents.

In total, 128 fractures were seen in the lateral midfacial region. The most common site of fractures was the zygomatic bone (68%), followed by the orbital floor (17%) and zygomatic arch (15%).

No trend could be detected in fracture patterns over the 11-years period of the study.

-Treatment

Mandibular fractures were mostly repaired by osteosynthesis (67%), with (49%) or without (18%) intermaxillary fixation, the proportion of osteosynthesis without intermaxillary fixation significantly (*P* < 0.01) increasing over time (from 13 ± 5 to 26 ± 1% in early and late 4-years groups, respectively). The remaining fractures were treated by intermaxillary fixation (33%). The lateral midfacial fractures were treated by reduction either alone (80%) or combined with internal fixation using plate (20%).

**Figure 7:**
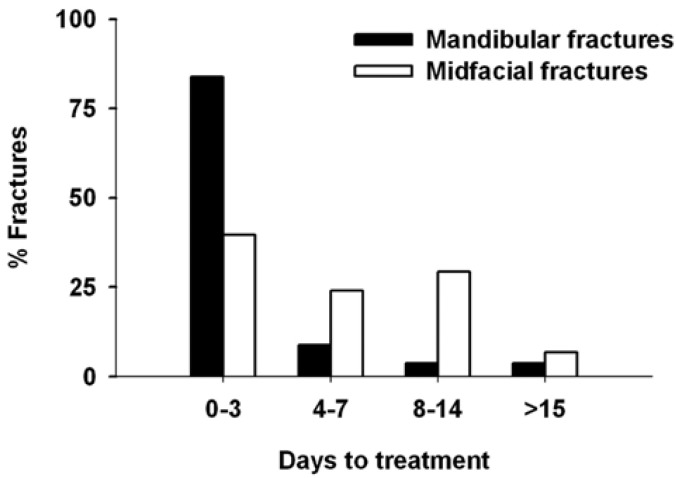
Time taken for repair of mandibular and lateral midfacial fractures.

The mean time between injury and surgery management was 3.6 ± 0.3 days (range 0-45 days) (Fig. 7). Most patients were treated within 3 days after injury (84%). But the delay of treatment was significantly (*P* < 0.001) shorter for mandibular fractures (2.4 ± 0.3 days, range 0-30 days) than for lateral midfacial fractures (6.4 ± 0.6 days, range 0-45 days). It has to be noted that this latter delay was not shortened for orbital floor and medial wall fractures with orbital soft tissue entrapment.

-Complications

In our series, complications occurred in 7 patients (2%). All complications involved the mandible: at the body (4 cases), symphysis (1 case) or both the body and angle (2 cases). Complications were: pseudoarthrosis (1 case) and exposed fixation material (6 cases) that required plate removal, two of which being due to infection. It has to be noted that material was removed in another patient, a boxer who had a fracture of the mandible body, according to his will. Age, gender, alcohol, tobacco, etiology and treatment of fractures were not associated with any risk to develop complications.

## Discussion

-Patients

A total of 364 patients with maxillofacial injuries were treated in the Oral and Maxillofacial Surgery Department of Clermont-Ferrand University Hospital between January 1997 and December 2007. Over this 11-years period, the incidence of maxillofacial fractures almost doubled. Surprisingly though, patient demographics remained rather steady. There was only an increase in tobacco use. Overall, the most common mechanisms of injury were interpersonal violence, traffic accidents and falls with traffic accidents reducing and falls increasing as a cause of fracture. About two-third of fractures involved the mandible. Most of these mandibular fractures were treated by osteosynthesis with or without intermaxillary fixation, with the proportion of the latter increasing over time. The incidence of complications remained very low.

-Demographic profile of patients

Maxillofacial fractures were more frequent in male (82%) than female (18%) patients. In addition, male (~32 years) were significantly younger than female (~48 years) patients. Similar gender and age predominance was previously noted (1). Higher prevalence of fractures in young men is probably due to exposure to numerous behavioral risks such as sport, driving and greater involvement in acts of violence (1,3). In addition, the mean age of female patients tended to increase, although not significantly, over the 11-years period of the study, suggesting that there was a trend towards increasing presentation of elderly female patients. This is consistent with falls increasing as a cause of fractures (see below). Such increased presentation of elderly female patients might account for the increasing number of female patients over time. Surprisingly though, there was no similar increase in the mean age of male patients, suggesting that the demographic profile of male patients has not changed over time.

-Mechanisms of injury

The causes of maxillofacial fractures vary depending on the geographic, demographic and socioeconomic characteristics of people. In most countries, traffic accidents are the main cause of maxillofacial injuries (1). Here, maxillofacial fractures were first due to interpersonal violence and then to traffic accidents, falls, and sport- and work-related accidents. It is interesting to note that the most recently published studies on maxillofacial fractures also consistently identify interpersonal violence as the most common etiology of maxillofacial fractures nowadays in developed countries (1,4,5). In addition, there were trends towards traffic accidents reducing and falls increasing as causes of maxillofacial fractures. Similar trends have been observed in the mechanisms of major traumatic injuries of New South Wales patients (6). Legislative changes and preventive measures involving seat belt and airbag use, as well as the reduction of drinking and driving, likely account for the reduced incidence of traffic accident-related facial injuries in some developed countries (1). High incidence of fall-related maxillofacial fractures in elderly people has also been reported (7-9). It likely results from the combined functional consequences of aging, systemic pathologies and the use of psychotropic drugs. Furthermore, elderly patients with osteoporosis more likely develop maxillofacial fractures following low-impact trauma (8), implying that osteoporosis is a risk factor for the development of maxillofacial fractures.

-Fractures pattern

As previously noted (1,4,10), maxillofacial fracture involve more frequently the mandible (~70%) than the middle third of the face (~30%). The position of the mandible at the forefront of the facial skeleton, its mobility and, thus, its lower bony support compared with maxilla (11), makes it more prone to trauma and fractures, alone or in association with other facial fractures, than maxilla.

The anatomic distribution and incidence of mandibular fractures are highly variable. In the present study, body fractures were the most common mandibular fracture (29%) followed by angle fractures (10%). In addition, combined body and angle fractures were also the most frequent double fractures (32%). Such high incidence of body fracture has been previously reported (3,11,12,13). It is noteworthy, however, that the angle (14,15), the body (16) or the symphysis (13) of the mandible were also found to be the most frequently affected sites.

Mandible body and angle are assumed to brake more easily than other mandibular regions because they are naturally weak areas. The mandible body region, lateral to the mental prominence, contains the canine fossa and the mental foramen. Similarly, several factors may contribute to the weakness of the mandibular angle. A first one is the presence of third molars as patients with impacted mandibular third molars exhibit a higher risk of angle fracture than patients without (17). Moreover, the region of the mandibular angle is thinner than more anterior or posterior mandibular regions (18). Together with the fact that the angle of the mandible is where there is an abrupt change in shape from horizontal to vertical rami, these factors can easily explain why fractures often occur in this location. The low incidence of condylar fractures in our study is likely related to our inclusion criteria. Indeed, we selected only patients who underwent surgical management under general anesthesia and the majority of patients with condylar fractures were treated with medical rehabilitation and monitoring.

The mandible fractures were mostly unifocal (60%) or bifocal (32%). Triple fractures were only occasional (8%). The average number of fractures per patient was 1.48. This number is higher than the 1.32 obtained by Le et al. (19) but similar to those re-ported by others (14,16). Interestingly, we found that the number of fractures per patient varied accordingly to the mechanism of injury. It was low (1.21) after work-related and high (1.64) after traffic accidents. Consistently, we also found that unifocal fractures were associated with interpersonal violence, while traffic accidents were often the cause of trifocal fractures. Others reporting a high number of fractures per patients similarly noted that traffic-accidents tend to cause a great number of fractures per patients because of the high impact force (14,16). Altogether, these findings suggest that the complexity fracture is associated with the energy of the trauma.

There were 128 patients with lateral midfacial fractures. The most common fracture was tripod or zygomaticomaxillary complex fractures (68%), so called because it involves separation of all three major attachments of the zygoma to the rest of the face, followed by fractures of the orbital floor (17%) and the zygomatic arch (15%), as reported previously (20,21).

-Treatment and complications

In the last years, plate osteosynthesis has become popular in managing maxillofacial fractures. This technique produces a stable anatomic reduction of the fragments, thus decreasing the risk of postoperative displacement of the fractured fragments, and does not require intermaxillary fixation in the postoperative period (22). It also allows immediate functional recovery, shortens the period of bone remodeling and consolidation of the fracture site and decreases the recovery period (22,23). In the present study, the majority of the mandibular fractures were treated by osteosynthesis with or without intermaxillary fixation. And the proportion of osteosynthesis without intermaxillary fixation increased over the 11-years period of the study. The remaining fractures were treated using the conservative technique of intermaxillary fixation alone. The lateral midfacial fractures were treated either by open reduction alone and internal fixation using plate or by open reduction.

In our series, very few patients (2%) developed complications compared with 3% to 30% elsewhere (24). It has to be noted that our complication rate might be underestimated since other complications, such as malocclusions or diplopia, were not listed in our records. All complications occurred in the mandibular body region, in line with previous evidence showing that mandibular fractures are more prone to complications than lateral midfacial ones (25). A possible explanation for the low rate of infection in the present study is that all patients underwent prophylactic antibiotic and a strict dental hygiene care plan during at least five days. Moreover, they underwent nasogastric feeding for five days after operation. Recent reviews (25,26) of the effects of prophylactic antibiotics in the treatment of mandibular fractures have led to the conclusion that such treatment can prevent infection. Moreover, one shot or one day administration appears to work equally well as or even better than a 7-days course. However, none of the reviewed studies were randomized controlled trial (25). Therefore, large randomized controlled trials are still needed to guide the clinical practice on postoperative complications of maxillofacial fractures.

The time between diagnosis and surgery management was, in average, 2 days for mandibular fractures, and 6 days for lateral midfacial fractures. However, most subjects underwent treatment within 3 days after injury. Surgical management of midfacial fractures is often delayed to allow reduction of the initial swelling in soft tissues. We did not observe any correlation between the day of treatment and complication rate. The effect of treatment delay on healing of fractures has been a subject of discussion (27). Whereas some studies failed to find any effect of treatment delay on fracture healing (28,29), others reported an increasing number of complications in case of delayed treatment (30,31). A recent systematic review of the literature on the relation between treatment delay and healing complications concluded that there is no strong evidence for either acute or delayed treatment of mandibular fractures in order to minimize healing complications (27). However, it is clear that fractures should be treated as early as possible to relieve patients from pain and discomfort.

## Conclusion

This retrospective study shows that the maxillofacial fractures most frequently occur in mandible of young men. Overall, the most common mechanisms of injury were interpersonal violence, traffic accidents and falls with traffic accidents reducing and falls increasing as a cause of fracture. There was a significant age and gender difference in etiology of fractures. Young male patients were preferentially victims of interpersonal violence and traffic and sport-related accidents, while middle-aged ones were of falls and work-related accidents. Middle-aged female patients were preferentially victims of traffic accidents and interpersonal violence, while older ones were of falls. The number of the fractures per patient varied accordingly to the mechanism of injury: low after work-related accidents and high after traffic accidents. The majority of the mandibular fractures were treated by osteosynthesis with or without intermaxillary fixation, with the proportion of osteosynthesis without intermaxillary fixation increasing over time. A low rate of postoperative infections was observed that may be related to a prophylactic antibiotic and a strict dental hygiene care plan.
